# Laparoscopic Sleeve Gastrectomy in a Morbidly Obese Patient with Myasthenia Gravis: A Review of the Management

**DOI:** 10.1155/2015/593586

**Published:** 2015-07-29

**Authors:** Megana Ballal, Tracey Straker

**Affiliations:** Albert Einstein College of Medicine, Montefiore Medical Center, Bronx, NY 10467, USA

## Abstract

Myasthenia gravis, a disorder of neuromuscular transmission, presents a unique challenge to the perioperative anesthetic management of morbidly obese patients. This report describes the case of a 27-year-old morbidly obese woman with a past medical history significant for myasthenia gravis and fatty liver disease undergoing bariatric surgery. Anesthesia was induced with intravenous agents and maintained with an inhalational and balanced intravenous technique. The nondepolarizing neuromuscular blocker Cisatracurium was chosen so that no reversal agents were given. Neostigmine was not used to antagonize the effects of Cisatracurium. The goal of this approach was to reduce the risk of complications such as postoperative mechanical ventilation. The anesthetic and surgical techniques used resulted in an uneventful hospital course. Therefore, we can minimize perioperative risks and complications by adjusting the anesthetic plan based on the patient's physiology and comorbidities as well as the pharmacology of the drugs.

## 1. Introduction

As the obesity epidemic grows and spreads worldwide, the prevalence of patients simultaneously carrying a diagnosis of myasthenia gravis is also steadily increasing. Consequently, more patients with complex comorbidities are presenting for surgery. Bariatric surgery has become an important treatment option for those with morbid obesity who have failed traditional weight-loss methods. Review of the literature reveals few reports of patients with morbid obesity and myasthenia gravis undergoing anesthesia and surgery [1–5]. A literature search was conducted on PubMed, Embase, and Web of Science using the keywords “myasthenia gravis,” “bariatric surgery,” “morbid^*∗*^,” and “obesity^*∗*^.”

Here we present the case of a morbidly obese patient with myasthenia gravis undergoing a laparoscopic sleeve gastrectomy. This report describes and discusses an anesthetic plan successfully used in a minimally invasive surgery in a patient with a complex past medical history.

## 2. Case Report

A 27-year-old woman with a body mass index of 40.9 kg/m^2^ presented for laparoscopic sleeve gastrectomy. Her past medical history included myasthenia gravis, which was diagnosed in July 2011 with primarily ocular symptoms. Other comorbidities were hypothyroidism, fatty liver disease, and anemia. Her only prior surgery was a thymectomy in December 2011. There were no anesthetic complications at that time. Her medication regimen included Prednisone 5 mg daily, Mestinon 60 mg TID, Imuran 250 mg daily, and Synthroid 137 mcg daily. The patient had no known drug allergies and denied alcohol, tobacco, or drug use.

The patient's cardiac functional status in metabolic equivalents (METS) was >4. Physical exam revealed no ptosis or diplopia. The patient was breathing comfortably on room air with no evidence of dysarthria or dysphagia. Airway assessment showed Mallampati score 3 with full range of motion of the neck and thyromental distance < 3 cm. Preoperative labs, including liver function tests, revealed no abnormalities. A prior electrocardiogram showed normal sinus rhythm at 68 beats per minute with mild left ventricular hypertrophy. A stress echocardiogram from the previous year demonstrated normal left ventricular systolic function with an ejection fraction of 60% and no signs of ischemia. The patient had a negative sleep study despite having a high risk of obstructive sleep apnea based on her body mass index as well as symptoms of snoring and tiredness.

On the morning of surgery, the only medication the patient had taken was Mestinon 60 mg. No anxiolytics, sedatives, opioids, or steroids were given during the preoperative phase. Standard monitoring and mask preoxygenation with 100% oxygen were provided once in the operating room. Anesthesia was induced with lidocaine 100 mg, propofol 200 mg, and Cisatracurium 14 mg.

Direct laryngoscopy and tracheal intubation were performed using a large Airtraq Avant guided intubation and a size 7.0 endotracheal tube, respectively. Anesthesia was maintained with fentanyl 150 mcg and desflurane to achieve a MAC 1.0. Neuromuscular blockade was titrated using a train-of-four (TOF) monitor with electrodes placed over the orbicularis oculi and corrugator supercilii muscles.

Laparoscopic sleeve gastrectomy was successfully performed over 90 minutes. The patient remained hemodynamically stable with minimal blood loss. Normosol 800 mL was given throughout the procedure. Desflurane was discontinued 10 minutes before the end of the procedure. Ondansetron was administered at the end of the case, and ketorolac was provided for pain management. No reversal agent was given for the residual neuromuscular blockade.

The patient awoke in the operating room 5 minutes after the desflurane was stopped. She indicated a desire to be extubated but was unable to follow commands or achieve appropriate tidal volumes. The patient was transferred while intubated to the postanesthesia care unit. She underwent successful tracheal extubation approximately 30 minutes after completion of the operation. Pain control was achieved with fentanyl patient-controlled analgesia (PCA) and intravenous Tylenol. Her postoperative course was unremarkable.

## 3. Discussion

Myasthenia gravis is a disorder of neuromuscular transmission characterized by fluctuating skeletal muscle weakness that worsens with repetitive use and improves with rest. There are three types of myasthenia gravis, neonatal, congenital, and autoimmune. The pathogenesis of the neonatal form is related to the transfer of maternal antibodies to the fetus, resulting in symptoms that last up to a few weeks after birth [6]. Congenital myasthenic syndrome is an inherited disorder caused by genetic defects at the neuromuscular junction [7]. The most common form of myasthenia gravis is autoimmune, with the two clinical subtypes being ocular and generalized.

Ocular myasthenia refers to weakness of the extraocular muscles and eyelids, resulting in diplopia and ptosis. The generalized form of the disease affects not only the ocular muscles, but also the respiratory, bulbar, and limb muscles to varying degrees. There is significant overlap between these two clinical forms, with many patients who initially present with ocular symptoms eventually developing generalized myasthenia [8].

Autoimmune myasthenia gravis is mediated by antibodies to proteins in the postsynaptic membrane of the neuromuscular junction. These include autoantibodies to the acetylcholine receptor or muscle-specific tyrosine kinase receptor. The presence of autoantibodies results in fewer available postsynaptic receptors and thus insufficient binding of acetylcholine to generate a muscle action potential. The age of onset has a bimodal distribution, affecting younger females and older males with increased frequency [9]. Diagnosis is typically confirmed with serologic testing and/or electrophysiologic studies, although a small percentage of patients are seronegative. Bedside methods such as the ice-pack test or edrophonium test can assist with making the diagnosis. Myasthenia gravis is often classified according to a modified Osserman scale or by categories determined by the Myasthenia Gravis Foundation of America [10]. Initial treatment for patients with mild to moderate symptoms is with an oral anticholinesterase such as pyridostigmine. If symptoms persist, thymectomy can be considered along with corticosteroids and other immunosuppressive agents [11].

Preoperative evaluation of this patient with myasthenia gravis involved assessing disease severity, especially bulbar and respiratory muscle function, and treatment regimen. Severe oropharyngeal or respiratory muscle weakness can even lead to a life-threatening condition known as myasthenic crisis. Myasthenic crisis either occurs spontaneously or is precipitated by certain factors such as infection or surgery [12]. Alternatively, cholinergic crisis may occur. Respiratory failure in cholinergic crisis is caused by excess cholinesterase inhibitors, which results in overstimulation with acetylcholine. The muscles, during cholinergic crisis, stop responding to acetylcholine and result in respiratory failure.

In 1980, Leventhal et al. analyzed the preoperative data of 24 myasthenic patients undergoing thymectomy in an effort to predict the need for postoperative mechanical ventilation. Four risk factors were identified: duration of disease > 6 years, history of chronic respiratory disease, pyridostigmine dose > 750 mg/day, and vital capacity < 2.9 liters [13]. However this scoring system has limited predictive value among those undergoing procedures other than thymectomy [14].

Alternate prognostic factors have been proposed since that time but are still under debate. The patient in this case did not exhibit any bulbar or respiratory symptoms prior to surgery.

Consequently, pulmonary function tests were not conducted preoperatively, and there was less concern about her ability to maintain a patent airway postoperatively.

The perioperative medication regimen, specifically pyridostigmine dosing, depends on the patient's severity of symptoms and psychological state as well as physician preferences. In order to develop an appropriate anesthetic plan, it is crucial to understand how myasthenic patients respond to certain drugs.

Neuromuscular blocking agents are classified as nondepolarizing (antagonist) or depolarizing (agonist). Myasthenic patients have increased sensitivity to nondepolarizing drugs [15]. Long-acting muscle relaxants are typically not used among this population. Use of short- and intermediate-acting agents can be monitored with electromyogram or mechanomyogram to assess the extent of neuromuscular transmission following stimulation. Response to electrical stimulation of a peripheral motor nerve is assessed differently depending on whether the block is nondepolarizing or depolarizing. With TOF stimulation in a nondepolarizing block where T4 < T3 < T2 < T1, the fade ratio of T4/T1 is important to determine the extent of the block. With a depolarizing block, T4 = T1 with TOF stimulation, meaning that there is no fade with the response. Various studies comparing the effective doses (ED95) of muscle relaxants for myasthenic patients and controls show increased sensitivity among those with myasthenia gravis. In this patient, Cisatracurium, a relatively expensive drug, was preferred over other nondepolarizing neuromuscular agents in this patient because it is degraded by Hofmann elimination. Therefore it is relatively safe to use in patients with liver or renal disease. Myasthenic patients are resistant to depolarizing agents such as succinylcholine. This is likely because there are less acetylcholine receptors for these neuromuscular blockers to act on [16].

It must be pointed out, at this juncture, that this management may differ from that of patients that do not have myasthenia gravis. In patients with normal physiology, you do not see resistance depolarizing muscle relaxants such as succinylcholine. Moreover, the concern for precipitation of a cholinergic crisis from too much anticholinesterase is nonexistent. The concern for precipitating a cholinergic crisis in the myasthenic patient was circumvented by giving a muscle relaxant, Cisatracurium, which does not need to be reversed by an anticholinesterase.

Nonmuscle relaxant techniques involving a combination of inhalational agents and intravenous anesthesia alone have also been described [17]. This case involving abdominal surgery was not amenable to that technique. With respect to the volatile anesthetics, sevoflurane has been shown to be potent and effective in myasthenic patients, as its low blood solubility allows for rapid elimination and recovery of neuromuscular transmission after discontinuation [18, 19]. As for the intravenous anesthetics, propofol and opioid analgesics have not been shown to significantly effect neuromuscular transmission. Yet there is a concern about respiratory depression with the use of opioids. This has led to the use of remifentanil, which has a short half-life allowing for easier titration during induction of anesthesia [20].

Reversal of neuromuscular blockade at the end of the procedure can occur spontaneously or with the use of certain agents such as neostigmine or, more recently, sugammadex [21]. In a nonmyasthenic patient, neostigmine is administered in order to increase the amount of acetylcholine at the neuromuscular junction and antagonize any residual effects of the NMBAs. Due to neostigmine's effect on the heart rate, this parasympathomimetic is given with glycopyrrolate, which blocks muscarinic receptors and thus prevents bradycardia. This combination of anticholinesterase and antimuscarinic agents was not administered in this case as there was a concern for precipitating a cholinergic crisis in a myasthenic patient. Therefore, the patient underwent spontaneous recovery and was extubated after meeting extubation criteria: ability to follow commands, stable vital signs with adequate respiratory mechanics, and reversal of neuromuscular blockade assessed by grip strength and head lift > 5 seconds.

While myasthenia gravis is a disease with several anesthetic implications, morbid obesity is a multisystem disorder with its own set of associated risks. Respiratory complications of obesity include obstructive sleep apnea and obesity hypoventilation syndrome. In addition to clinical symptoms such as morning headaches and daytime somnolence, these conditions can induce important pathophysiologic changes. Examples of altered physiology with obesity are hypoxemia and hypercapnia, eventually leading to pulmonary hypertension. Patients with morbid obesity also have reduced expiratory reserve volume and functional residual capacity. This is caused by decreased chest wall compliance due to excess thoracic adipose tissue and reduced lung compliance due to small airway collapse, increased pulmonary blood volume, and displacement of abdominal contents [22]. This case illustrates the considerations that must be taken into account when giving an anesthetic to a patient with myasthenia gravis and morbid obesity. Alterations in anesthetic management are advised based on the severity of the patient's symptoms, resistance, and sensitivity to anesthetic agents as well as the potential to precipitate a cholinergic crisis. As anesthetic agents continue to evolve, management of myasthenia gravis in the morbidly obese patient needs to be continually addressed.

We advocate that, in the management of bariatric myasthenic patients, some key principles should be considered. They are as follows:

1. If muscle relaxation is needed, consider the use of Cisatracurium.
2. Use ultrashort acting opioids, such as remifentanil, as well as multimodal analgesic techniques for post-op pain management.
3. Be mindful that this patient population may have risk factors for respiratory sensitivity due to myasthenia gravis as well as obstructive sleep apnea.
4. Be prepared for postoperative mechanical ventilation and do not rush the extubation process.

## References

[1] Jahr J. S., Bjerke R. J. (1991). Intrathecal morphine for thymectomy in a morbidly obese patient with myasthenia gravis. *The Journal of the Louisiana State Medical Society*.

[2] Schumann R., Tarnoff M., Siddiqui Z. I. (2004). Minimally invasive gastric bypass in a morbidly obese patient with myasthenia gravis. *Obesity Surgery*.

[3] Arias F., Szomstein S., Carrodeguas L. (2005). Myasthenia gravis improvement after laparoscopic Roux-en-Y gastric bypass. *Obesity Surgery*.

[4] Ui T., Hosoya Y., Kurashina K. (2011). Laparoscopic-assisted proximal gastrectomy in an obese patient with gastric cancer and myasthenia gravis. *Surgical Laparoscopy, Endoscopy & Percutaneous Techniques*.

[5] Gagné D. J., Papasavas P. K., Dovec E. A., Urbandt J. E., Caushaj P. F. (2009). Effect of immunosuppression on patients undergoing bariatric surgery. *Surgery for Obesity and Related Diseases*.

[6] Papazian O. (1992). Transient neonatal myasthenia gravis. *Journal of Child Neurology*.

[7] Engel A. G., Ohno K., Sine S. M. (2003). Congenital myasthenic syndromes: progress over the past decade. *Muscle and Nerve*.

[8] Sommer N., Melms A., Weller M., Dichgans J. (1993). Ocular myasthenia gravis—a critical review of clinical and pathophysiological aspects. *Documenta Ophthalmologica*.

[9] Drachman D. B. (1978). Myasthenia gravis. *The New England Journal of Medicine*.

[10] Jaretzki A., Barohn R. J., Ernstoff R. M. (2000). Myasthenia gravis: recommendations for clinical research standards. *The Annals of Thoracic Surgery*.

[11] Drachman D. B. (1978). Myasthenia gravis. *The New England Journal of Medicine*.

[12] Berrouschot J., Baumann I., Kalischewski P., Sterker M., Schneider D. (1997). Therapy of myasthenic crisis. *Critical Care Medicine*.

[13] Leventhal S. R., Orkin F. K., Hirsh R. A. (1980). Prediction of the need for postoperative mechanical ventilation in myasthenia gravis. *Anesthesiology*.

[14] Grant R. P., Jenkins L. C. (1982). Prediction of the need for postoperative mechanical ventilation in myasthenia gravis: thymectomy compared to other surgical procedures. *Canadian Anaesthetists Society Journal*.

[15] Blichfeldt-Lauridsen L., Hansen B. D. (2012). Anesthesia and myasthenia gravis. *Acta Anaesthesiologica Scandinavica*.

[16] Abel M., Eisenkraft J. B. (2002). Anesthetic implications of myasthenia gravis. *Mount Sinai Journal of Medicine*.

[17] Della Rocca G., Coccia C., Diana L. (2003). Propofol or sevoflurane anesthesia without muscle relaxants allow the early extubation of myasthenic patients. *Canadian Journal of Anesthesia*.

[18] Nishi M., Nakagawa H., Komatsu R., Natsuyama T., Tanaka Y. (1993). Neuromuscular effects of sevoflurane in a patient with myasthenia gravis. *Journal of Anesthesia*.

[19] Kiran U., Choudhury M., Saxena N., Kapoor P. (2000). Sevoflurane as a sole anaesthetic for thymectomy in myasthenia gravis. *Acta Anaesthesiologica Scandinavica*.

[20] Stephenson L., Tkachenko I., Shamberger R., Seefelder C. (2011). Anesthesia for patients undergoing transsternal thymectomy for juvenile myasthenia gravis. *Saudi Journal of Anaesthesia*.

[21] Rudzka-Nowak A., Piechota M. (2011). Anaesthetic management of a patient with myasthenia gravis for abdominal surgery using sugammadex. *Archives of Medical Science*.

[22] Parameswaran K., Todd D. C., Soth M. (2006). Altered respiratory physiology in obesity. *Canadian Respiratory Journal*.

